# The Effect of Empowering Education Combined With Mindfulness Meditation Training on Negative Emotion and Quality of Life in Patients With Inflammatory Bowel Disease

**DOI:** 10.3389/fnbeh.2022.901696

**Published:** 2022-06-02

**Authors:** Wei-Zhen Xi, Chong-Wu Xu, Ling-Ling Wang

**Affiliations:** Department of Gastroenterology, The First Affiliated Hospital of Wannan Medical College, Wuhu, China

**Keywords:** empowerment education, mindfulness meditation training, IBD, anxiety, quality of life

## Abstract

**Objective:**

To investigate the effect of empowerment education combined with mindfulness meditation training on negative emotions and quality of life in patients with inflammatory bowel disease (IBD).

**Methods:**

A total of 40 patients with IBD were selected and divided into experimental group and control group according to the random number table method, 20 cases in each group. The patients in the control group were treated with conventional nursing methods. The patients in the experimental group used empowerment education combined with mindfulness meditation training. Changes in mood and quality of life were compared between the two groups at admission and 3 months after discharge.

**Results:**

There was no significant difference in SAS score and quality of life score between the two groups. After authorization education combined with mindfulness meditation training, the SAS score of the experimental group was significantly lower than that of the control group (*P* < 0.01). The score of quality of life in the experimental group was significantly higher than that in the control group (*P* < 0.01).

**Conclusion:**

Empowerment education combined with mindfulness meditation can effectively improve the negative emotions and quality of life of patients with inflammatory bowel disease, and benefit the patients in many aspects.

## Introduction

Inflammatory bowel disease (IBD) is a group of chronic, relapsing, and non-specific inflammatory diseases involving the gut (Guan, 2019; Dash et al., 2021). Inflammatory bowel disease is clinically divided into ulcerative colitis (UC) and Crohn’s disease (CD) (Lamb et al., 2019). In recent years, the incidence rate of IBD has increased significantly. The incidence rate of low incidence rate in Eastern Europe and Asia is also increasing. The prevalence rates of UC and Cd in China are (120–200)/100,000 and (50–200)/100,000, respectively (Ng et al., 2016). IBD patients are more prone to negative emotions such as anxiety and depression, and their quality of life is significantly reduced (Rogler et al., 2021). Long-term negative emotions will not only aggravate the patient’s condition, but also lead to new psychosomatic symptoms, reduce the patient’s quality of life, affect the treatment of inflammatory bowel disease, and increase the risk of relapse (Atanasova et al., 2021). Mindfulness meditation is a form of body and mind based intervention to guide patients to focus their personal attention on the current experience with an open, curious and receptive attitude (Gok Metin et al., 2019). Studies have shown that mindfulness meditation has good effects in reducing nausea, pain, fatigue, strengthening strategies to deal with stress events and mental health (Boyle et al., 2017; Ruihong et al., 2020). As one of the means of treatment implementation, routine health education plays a positive role in promoting patients’ physical and mental health, but it has some defects, such as short intervention time, discontinuous process, incomplete content and so on (Darnall et al., 2021). Empowerment education is a new educational model with empowerment and self-management as the core and educational process as the center. Advocate patients by stimulating self-management motivation, actively pay attention to their own health, clarify self-management responsibilities, promote behavioral changes, and actively implement self-behavioral care (Kim and You, 2017). At present, this model is widely used in chronic disease health education and has achieved remarkable results (Guo et al., 2020). However, there are few reports on the clinical therapeutic effect of empowerment education combined with mindfulness meditation training. Therefore, this study aims to explore the implementation of empowerment education combined with mindfulness meditation training to help patients with inflammatory bowel disease improve their negative emotions and quality of life.

## Objects and Methods

### Research Object

**FIGURE 1:**
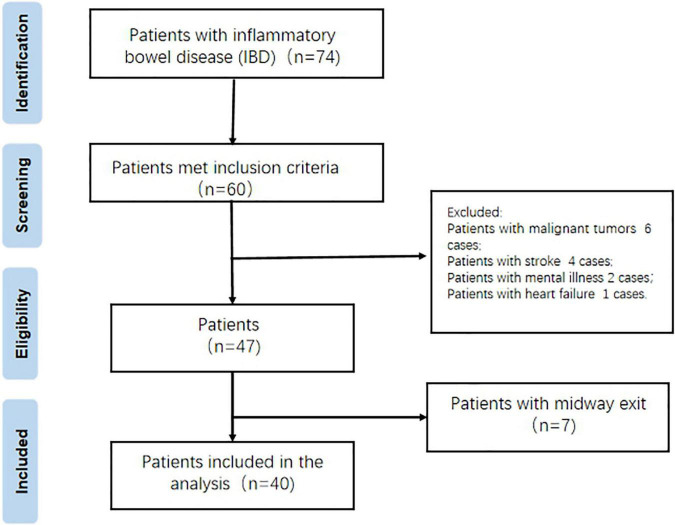
The flow chart.

Forty patients with IBD admitted to the Department of Gastroenterology of the First Affiliated Hospital of Wannan Medical College from January 2018 to May 2020 were selected (Figure 1). According to the random number table method, they were divided into experimental group and control group, 20 cases in each group.

Inclusion criteria: (1) Meet the diagnostic criteria of the Third European Guidelines for the Diagnosis and Treatment of UC and CD (Magro et al., 2017); (2) Clear consciousness, certain understanding and writing skills, and barrier free communication; (3) Informed consent and sign the informed consent form.

Exclusion criteria: (1) Patients with other serious systemic diseases (such as malignant tumor, stroke, heart failure, etc.); (2) Patients with previous or current mental diseases; (3) Those who have received formal psychotherapy. This study is a cross-sectional study and has been approved by the hospital ethics committee.

### Methods

#### Control Group

The control group adopted routine nursing methods: simple understanding of disease-related knowledge, treatment, exercise and dietary guidance, and psychological comfort to patients with poor mood.

#### Experimental Group

The experimental group adopted the nursing method of empowerment education combined with mindfulness meditation training: each intervention was arranged by the head nurse with higher qualifications and completed by professionally trained responsible nurses. The training methods were mainly empowerment education and the teaching of mindfulness meditation training theory. Explain the content and related matters to the nursing staff, distribute relevant materials, and give those who scored more than 80 points in the written test to complete the nursing mode of authorized education combined with mindfulness meditation training, and the nurse in charge of the patient will notify and arrange. The specific process is as follows:

➀ Establish the problem.

On the first day of admission, the patients’ physical and psychological status were comprehensively evaluated, their needs for relevant disease knowledge were evaluated, their worries about treatment were understood, their cognitive level of disease treatment plan and self-care ability were clarified, and their negative emotions were evaluated.

➁ Mindfulness meditation training.

Responsible nurses actively communicate with patients, observe whether their emotions have changed during the conversation, understand the causes of patients’ psychological pressure, and give psychological counseling. Explain the contents, methods and precautions of mindfulness meditation training. First, direct mindfulness breathing. Feel the feeling of nasal breath flowing in and out, and be aware of the feeling that breathing brings to this part, so that patients do not count breath, do not control and regulate breath. Second, guide the body scan. The responsible nurse guides the patients to choose a comfortable position, close their eyes after relaxing, play soft and soothing music, imagine a soft beam, guide the patients to pay attention to the imaginary beam, start from the top of the head, slowly move down, and scan the whole body from head to foot. After mastering the training methods, the patients were trained once a day in the afternoon and before going to bed at night, 30 min per time, more than 5 days per week for 3 consecutive months.

➂ Set goals.

Guide patients to set practical goals, including diet, exercise, living habits, emotional control, treatment, and complication prevention, so that patients can actively cooperate and participate in self-care management.

➃ Plan formulation and implementation.

It mainly includes how to master the relevant knowledge of disease, self-care skills, mindfulness meditation training methods and exercise planning methods, time frequency and so on. In the process of implementation, it is necessary to communicate with patients in time to understand the implementation situation and the problems encountered in the implementation process, and timely solve them. It is necessary to affirm the efforts of patients in the implementation process, and enhance the confidence of patients to overcome the disease.

➄ Observation, follow-up and evaluation.

The patient insisted on practicing for 3 months after discharge. They can communicate with patients by telephone twice a week on Tuesday and Friday through telephone follow-up, SMS push and face-to-face follow-up, so as to understand the current mental, psychological and physical conditions, treatment compliance, diet and exercise of patients. If the patient fails to answer the phone in time, they can push short messages to ask about the patient’s current situation, and answer questions. At the same time, they can regularly push the relevant knowledge of inflammatory bowel disease. Face to face follow-up was conducted every half a month to evaluate the mastery of health education, the effect of mindfulness meditation training and the degree of disease control.

### Evaluation Tools

#### Self Rating Anxiety Scale

It adopts 4-level scoring method. The evaluation criteria are as follows: (1) point means no or little time, (2) points means a small part of time, (3) points means a considerable amount of time, and (4) points means most or all of the time. Each scale has 20 items for evaluation. After the evaluation, all items are added up to get a rough score, which is multiplied by 1.25, Take the integral part to get the final score. The higher the score, the heavier the negative emotion (Wang et al., 2021).

#### Quality of Life Questionnaire for Inflammatory Bowel Disease

Applying this questionnaire based on the literature (Yarlas et al., 2020), there are 32 questions in total, each with an answer ranging from 1 to 7. The scoring range is 32–224 points. The higher the score, the better the quality of life.

#### Medication Compliance Scale

Morisky medication compliance scale was used (Li and Ying, 2015). It is composed of five questions. Each question has four options (impossible at all, occasional, basic, and complete) and is given 1∼4 points, respectively, with a total score of 5∼20 points. The higher the score, the better the patient’s medication compliance.

#### Lifestyle Compliance Questionnaire

The lifestyle compliance questionnaire was used, with a total of 9 items (Dubinsky et al., 2021) (avoid eating foods that induce IBD, follow the doctor’s advice to quit smoking, follow the doctor’s advice to quit drinking, follow the doctor’s advice to eat healthily, follow the doctor’s advice to avoid overeating, follow the doctor’s advice to avoid overwork, follow the doctor’s advice to avoid bad emotions, follow the doctor’s advice to exercise properly, and follow the doctor’s advice to review regularly). Likert grade 5 scoring method, with 1∼5 points, respectively, means “can’t do it at all,” “can do it occasionally,” “can do it sometimes,” “can do it basically,” and “can do it completely.” The higher the score, the better the patient’s lifestyle compliance.

### Statistical Methods

Statistical software SPSS 19.0 was used to process and analyze the data. The measurement data was expressed by mean ± standard deviation($\bar{x}$ s), *t*-test was used, and the count data was analyzed by χ^2^-test, *P* < 0.05 for the difference was statistically significant.

## Results

### Comparison of Baseline Data Between the Two Groups

There was no significant difference in baseline data of gender, age, marital status, education level, and work status between the two groups (*P* > 0.05), which was comparable (see Table 1).

**TABLE 1:** Comparison of general information of patients with IBD between the two groups.

Group	Case	Gender (case)		Age	Marriage		Education level			Work	
		Male	Female	(years, $\bar{x}$ ± s)	Yes	No	<High school	High school or equivalent	≥College	Yes	No
Experimental	20	14	6	30.1 ± 16.1	12	8	3	6	11	13	7
Control	20	13	7	30.8 ± 13.4	13	7	3	7	10	14	6
t/χ^2^		0.085		0.025	0.085		0.111			0.044	
*P*		0.933		0.980	0.933		0.912			0.965	

### The Comparison of Self Rating Anxiety Scale Evaluation Results Between the Two Groups at Admission and 3 Months After Discharge

There was no significant difference in Self rating Anxiety Scale scores between the two groups at admission (*P* > 0.05). The SAS scores of the experimental group were significantly lower than those of the control group 3 months after discharge (*P* < 0.01) (Table 2).

**TABLE 2:** Comparison of SAS scores of two groups of patients with IBD at admission and 3 months after discharge ($\bar{x}$ ± s).

Group	*N*	On admission	3 months after discharge	*t*	*P*
Experimental	20	57.88 ± 6.16	45.32 ± 5.92	7.351	<0.001
Control	20	56.52 ± 4.95	55.28 ± 5.02	0.879	0.384
*t*		0.861	6.413		
*P*		0.393	<0.001		

### The Comparison of Quality of Life Scores Between the Two Groups at Admission and 3 Months After Discharge

There was no significant difference in the scores of quality of life between the two groups at admission (*P* > 0.05). The scores of quality of life in the experimental group were significantly higher than those in the control group 3 months after discharge (*P* < 0.01) (Table 3).

**TABLE 3:** Comparison of quality of life scores of two groups of patients with IBD at admission and 3 months after discharge ($\bar{x}$ ± s).

Group	Cases	At admission	3 months after discharge	*t*	*P*
Experimental	20	136.12 ± 14.43	166.72 ± 14.30	7.531	<0.001
Control	20	135.04 ± 13.61	140.28 ± 11.97	1.446	0.154
*t*		0.272	7.090		
*P*		0.787	<0.001		

### Comparison of Medication Compliance and Life Compliance Scores Between the Two Groups at Admission and 3 Months After Discharge

There was no significant difference in the scores of medication compliance and life compliance between the two groups at admission (*P* > 0.05), but the scores of medication compliance and life compliance in the study group were higher than those in the control group 3 months after discharge, and the difference was statistically significant (*P* < 0.05), which became more and more obvious with the extension of time (see Tables 4, 5).

**TABLE 4:** Comparison of medication compliance scores between the two groups of IBD patients at admission and 3 months after discharge (x ± s).

Group	*N*	On admission	3 months after discharge	*t*	*P*
Experimental	20	17.51 ± 1.67	16.92 ± 1.30		
Control	20	17.14 ± 1.61	15.38 ± 1.47		
*t*		1.272	7.149		
*P*		0.237	<0.001		

**TABLE 5:** Comparison of life compliance scores between the two groups of IBD patients at admission and 3 months after discharge (x ± s).

Group	*N*	On admission	3 months after discharge	*t*	*P*
Experimental	20	40.51 ± 4.67	38.72 ± 7.30		
Control	20	35.04 ± 13.61	33.38 ± 8.77		
*t*		0.272	3.809		
*P*		0.457	<0.001		

## Discussion

### Empowerment Education Combined With Mindfulness Meditation Training Can Effectively Improve the Negative Emotions of Patients

The young and middle-aged patients with inflammatory bowel disease account for the main part. Due to repeated illness, long course of disease, and long-term suffering from the disease, the social function of patients is seriously damaged. In

addition, the medical expenses caused by long-term treatment are easy to increase the economic burden of families, which makes patients have different degrees of anxiety, depression and other negative emotions (Abautret-Daly et al., 2018). Negative emotions and the occurrence and development of inflammatory bowel disease will affect each other, which can affect the secretion of intestinal hormones and intestinal motility by changing the hypothalamus pituitary adrenal axis or affecting the intestinal flora, aggravate the clinical symptoms of patients with inflammatory bowel disease and increase the possibility of recurrence (Lester and Murrell, 2021). Therefore, for patients with inflammatory bowel disease, clinical attention should be paid to psychological intervention. In addition to the traditional drug therapy, mindfulness, as a training method of self-regulation of psychological stress, has gradually been concerned in clinical practice. Mindfulness training can help patients accept the attitude of non-judgment, deal with physical discomfort, reduce the psychological pressure of patients, and reduce the experience of negative emotions through breathing, body scanning, and other exercises (Barnhofer et al., 2021). The possible mechanism is: mindfulness meditation training can make the central nervous system, autonomic nervous system and mental state interact and promote each other, and in a good state of coordination, so as to effectively relieve stress, resist stress, and promote physical and mental health (Ostromohov et al., 2021). Integrating mindfulness meditation training into empowerment education can make patients relax, cultivate a peaceful state of mind, promote the change of patients’ behavior, adjust their emotions, improve their understanding of diseases, actively participate in disease management, set goals and make plans together with doctors and nurses, and when the set goals are achieved one by one, their confidence in overcoming diseases will be enhanced, Disease control and improvement will also improve the negative emotions of patients. The results showed that the SAS score of the experimental group was lower than that of the control group 3 months after discharge, and the difference was statistically significant (*P* < 0.01), indicating that the application of empowerment education combined with mindfulness meditation training can effectively improve the negative emotions of patients. This is the same result reported by some scholars (Zare et al., 2020; Kafaei-Atrian et al., 2022).

### Empowerment Education Combined With Mindfulness Meditation Training Can Improve the Quality of Life of Patients

In recent years, the incidence rate is higher and higher, disease relapse, gastrointestinal dysfunction, abdominal pain, diarrhea, mucus and bloody stools and other long-term physical discomfort brings anxiety and depression to patients, and their self-efficacy is reduced, which seriously affects the quality of life. Empowerment education is a new type of health education mode, which has strong pertinence. It can directly understand the patients’ needs for disease-related knowledge and their emotional state by guiding, establish the problems we need to solve, and then solve the existing problems, Nurses and patients work together to make plans and determine goals (Gu et al., 2019). The main reason is that traditional health education is a form of indoctrination and preaching health education. Patients only passively receive information, and patients only generalize the content of health education. Therefore, the effect of health education is limited (Ewais et al., 2020). The simple theoretical knowledge cannot effectively improve the situation of patients, especially the psychological care of patients. While empowering educators pay attention to explaining the knowledge of IBD to patients, they can stimulate the internal potential of patients and further improve the enthusiasm and initiative of patients. Its core is to let patients clarify the importance of self-management, actively formulate goals and act toward goals. Active action by patients can better carry out drug treatment and control diet, so as to improve the quality of life of patients.

During the intervention, mindfulness meditation training was combined. Mindfulness can improve the psychosocial adaptability of patients, improve the way of coping with diseases, and effectively improve the quality of life of patients (Morisky et al., 1986). At the same time, do not relax the intervention after discharge, continue to follow up regularly after discharge, through telephone follow-up, face-to-face follow-up and other forms to understand the patient’s diet, exercise, treatment compliance, emotional regulation, and other related content after discharge. Through empowerment education combined with mindfulness meditation training, strengthen internal factors, help patients reduce the repeated thinking of negative emotion cognition, enhance positive emotion experience, improve patients’ attention and executive control ability, promote patients to actively cooperate with treatment, and form positive guidance. In this study, although the IBDQ score of the control group also improved to a certain extent, the improvement was far less obvious than that of the experimental group, suggesting that the use of empowerment education combined with mindfulness meditation training can improve the quality of life of patients with inflammatory bowel disease.

Through this study, patients can carry out psychological adjustment and Health Empowerment, improve their self-efficacy, and actively participate in self-disease management, Improve the quality of life of patients. However, this study only selects IBD patients from a class III class a hospital as the research object, and it is a cross-sectional survey. The representativeness and inference of the results may be insufficient. In the future, we can expand the scope of investigation and implement longitudinal follow-up study to further explore the therapeutic effect of this method.

## Conclusion

To sum up, by applying empowerment education combined with mindfulness meditation training to patients with inflammatory bowel disease, it can effectively improve the patients’ knowledge of inflammatory bowel disease, enable patients to correctly understand the disease, enhance their awareness of active participation, regulate their own emotions, reduce the generation of negative emotions, adhere to the self-management of the disease, and improve the quality of life of patients.

## Data Availability Statement

The original contributions presented in the study are included in the article/supplementary material, further inquiries can be directed to the corresponding author/s.

## Ethics Statement

This study was conducted in accordance with the Declaration of Helsinki and approved by the First Affiliated Hospital of Wannan Medical College. All participants signed an informed consent form for inclusion in the study.

## Author Contributions

W-ZX conceived of the study. C-WX designed the study. L-LW drafted the manuscript. All authors read and approved the final manuscript.

## Conflict of Interest

The authors declare that the research was conducted in the absence of any commercial or financial relationships that could be construed as a potential conflict of interest.

## Publisher’s Note

All claims expressed in this article are solely those of the authors and do not necessarily represent those of their affiliated organizations, or those of the publisher, the editors and the reviewers. Any product that may be evaluated in this article, or claim that may be made by its manufacturer, is not guaranteed or endorsed by the publisher.

## References

[B1] Abautret-Daly Á Dempsey E. Parra-Blanco A. Medina C. Harkin A. (2018). Gut-brain actions underlying comorbid anxiety and depression associated with inflammatory bowel disease. *Acta Neuropsychiatr.* 30 275–296. 10.1017/neu.2017.3 28270247

[B2] Atanasova K. Lotter T. Reindl W. Lis S. (2021). Multidimensional assessment of interoceptive abilities, emotion processing and the role of early life stress in inflammatory bowel diseases. *Front. Psychiatry* 12:680878. 10.3389/fpsyt.2021.680878 34248716 PMC8264143

[B3] Barnhofer T. Reess T. J. Fissler M. Winnebeck E. Grimm S. Gärtner M. (2021). Effects of mindfulness training on emotion regulation in patients with depression: reduced dorsolateral prefrontal cortex activation indexes early beneficial changes. *Psychosom. Med.* 83 579–591. 10.1097/PSY.0000000000000955 34213860

[B4] Boyle C. C. Stanton A. L. Ganz P. A. Crespi C. M. Bower J. E. (2017). Improvements in emotion regulation following mindfulness meditation: effects on depressive symptoms and perceived stress in younger breast cancer survivors. *J. Consult. Clin. Psychol.* 85 397–402. 10.1037/ccp0000186 28230391 PMC5364040

[B5] Darnall B. D. Roy A. Chen A. L. Ziadni M. S. Keane R. T. You D. S. (2021). Comparison of a single-session pain management skills intervention with a single-session health education intervention and 8 sessions of cognitive behavioral therapy in adults with chronic low back pain: a randomized clinical trial. *JAMA Netw. Open* 4:e2113401. 10.1001/jamanetworkopen.2021.13401 34398206 PMC8369357

[B6] Dash K. R. Panda C. Das H. S. Mishra D. Behera S. K. Parida P. K. (2021). Association of vitamin d level with disease severity and quality of life in newly diagnosed patients of ulcerative colitis: a cross-sectional analysis. *Cureus* 13:e16481. 10.7759/cureus.16481 34430097 PMC8375454

[B7] Dubinsky M. C. Dotan I. Rubin D. T. Bernauer M. Patel D. Cheung R. (2021). Burden of comorbid anxiety and depression in patients with inflammatory bowel disease: a systematic literature review. *Expert Rev. Gastroenterol. Hepatol.* 15 1–13. 10.1080/17474124.2021.1911644 34130572

[B8] Ewais T. Begun J. Kenny M. Headey A. Tefay M. Kisely S. (2020). Mindfulness-based cognitive therapy experiences in youth with inflammatory bowel disease and depression: findings from a mixed methods qualitative study. *BMJ Open* 10:e041140. 10.1136/bmjopen-2020-041140 33148766 PMC7643511

[B9] Gok Metin Z. Karadas C. Izgu N. Ozdemir L. Demirci U. (2019). Effects of progressive muscle relaxation and mindfulness meditation on fatigue, coping styles, and quality of life in early breast cancer patients: an assessor blinded, three-arm, randomized controlled trial. *Eur. J. Oncol. Nurs.* 42 116–125. 10.1016/j.ejon.2019.09.003 31520865

[B10] Gu P. Lu S. Li Y. Zheng L. Li J. (2019). Type.2 diabetes mellitus, hypoglycemia fear and dietary behavior compliance. *J. Nurs.* 34 25–28.

[B11] Guan Q. A. (2019). Comprehensive review and update on the pathogenesis of inflammatory bowel disease. *J. Immunol. Res.* 2019 7247238. 10.1155/2019/7247238 31886308 PMC6914932

[B12] Guo X. Jia H. Zhang L. Ge X. Qu R. Dong H. (2020). Health empowerment intervention for elderly patients with chronic diseases based on community family doctor system. *J. Nurs.* 35 97–100.

[B13] Kafaei-Atrian M. Sadat Z. Nasiri S. Izadi-Avanji F. S. (2022). The effect of self-care education based on self-efficacy theory, individual empowerment model, and their integration on quality of life among menopausal women. *Int. J. Community Based Nurs. Midwifery* 10 54–63. 10.30476/IJCBNM.2021.86814.1370 35005041 PMC8724726

[B14] Kim S. H. You H. S. (2017). [The effects of an empowerment education program for kidney transplantation patients]. *J. Korean Acad. Nurs.* 47 445–455. 10.4040/jkan.2017.47.4.445 28894067

[B15] Lamb C. A. Kennedy N. A. Raine T. Hendy P. A. Smith P. J. Limdi J. K. (2019). British Society of Gastroenterology consensus guidelines on the management of inflammatory bowel disease in adults. *Gut* 68(Suppl. 3) s1–s106. 31562236 10.1136/gutjnl-2019-318484 PMC6872448

[B16] Lester E. G. Murrell A. R. (2021). An experimental study of mindfulness and acceptance-based skills for internalized ageism in older adults and college students. *Aging Ment. Health* 19 1–8. 10.1080/13607863.2021.1950613 34281430

[B17] Li W. Ying Z. (2015). Investigation and analysis of influencing factors of treatment compliance in patients with inflammatory bowel disease. *Qilu J. Nurs.* 21 6–8. 10.1186/s13054-016-1208-6 27885969 PMC5493079

[B18] Magro F. Gionchetti P. Eliakim R. Ardizzone S. Armuzzi A. Barreiro-de Acosta M. (2017). Third European evidence-based consensus on diagnosis and management of ulcerative colitis. part 1: definitions, diagnosis, extra-intestinal manifestations, pregnancy, cancer surveillance, surgery, and ileo-anal pouch disorders. *J. Crohns Colitis* 11 649–670. 10.1093/ecco-jcc/jjx008 28158501

[B19] Morisky D. E. Green L. W. Levine D. M. (1986). Concurrent and predictive validity of a self-reported measure of medication adherence. *Med Care* 24 67–74. 10.1097/00005650-198601000-00007 3945130

[B20] Ng W. K. Wong S. H. Ng S. C. (2016). Changing epidemiological trends of inflammatory bowel disease in Asia. *Intest Res.* 14 111–119. 10.5217/ir.2016.14.2.111 27175111 PMC4863044

[B21] Ostromohov G. Fibelman M. Hirsch A. Ron Y. Cohen N. A. Kariv R. (2021). Assessment of patients’ understanding of inflammatory bowel diseases: development and validation of a questionnaire. *United Eur. Gastroenterol. J.* 10 104–114. 10.1002/ueg2.12182 34939350 PMC8830304

[B22] Rogler G. Singh A. Kavanaugh A. Rubin D. T. (2021). Extraintestinal manifestations of inflammatory bowel disease: current concepts, treatment, and implications for disease management. *Gastroenterology* 161 1118–1132. 10.1053/j.gastro.2021.07.042 34358489 PMC8564770

[B23] Ruihong Z. Huiqiong Y. Jiaxin L. Cuilan X. Jie P. Ruyi L. (2020). Intervention effect of rational emotional therapy on negative emotion of patients with recurrent hospitalized advanced schistosomiasis. *Chinese J. Schistosomiasis Control* 32 308–310. 10.16250/j.32.1374.2020098 32468797

[B24] Wang Y. Sun K. Zhang W. Zhang H. Wang C. (2021). Pain and psychological distress: effect of microvascular decompression on sleep disorders and obsessions in trigeminal neuralgia. *J. Neurol. Surg. B Skull Base* 82(Suppl 3) e285–e294. 10.1055/s-0039-3402040 34306951 PMC8289503

[B25] Yarlas A. Maher S. Bayliss M. Lovley A. Cappelleri J. C. Bushmakin A. G. (2020). The inflammatory bowel disease questionnaire in randomized controlled trials of treatment for ulcerative colitis: systematic review and meta-analysis. *J. Patient Cent. Res. Rev.* 7 189–205. 10.17294/2330-0698.1722 32377552 PMC7197888

[B26] Zare F. Ghafari M. Ramezankhani A. Kamran B. L. Kavoosi A. (2020). Identifying dimensions of empowerment in patients with inflammatory bowel disease: a qualitative study. *Health Educ. Res.* 35 637–647. 10.1093/her/cyaa023 32995862

